# The Interplay between Vitamin D, Exposure of Anticholinergic Antipsychotics and Cognition in Schizophrenia

**DOI:** 10.3390/biomedicines10051096

**Published:** 2022-05-09

**Authors:** Arnim Johannes Gaebler, Michelle Finner-Prével, Federico Pacheco Sudar, Felizia Hannah Langer, Fatih Keskin, Annika Gebel, Jana Zweerings, Klaus Mathiak

**Affiliations:** 1 Department of Psychiatry, Psychotherapy and Psychosomatics, Faculty of Medicine, RWTH Aachen University, 52062 Aachen, Germany; mfinner@ukaachen.de (M.F.-P.); fpachecosuda@ukaachen.de (F.P.S.); flanger@ukaachen.de (F.H.L.); annika.gebel@rwth-aachen.de (A.G.); jzweerings@ukaachen.de (J.Z.); kmathiak@ukaachen.de (K.M.); 2 JARA—Translational Brain Medicine, 52062 Aachen, Germany; 3 Institute of Physiology, Faculty of Medicine, RWTH Aachen University, 52062 Aachen, Germany; 4 Klinik Königshof, 47807 Krefeld, Germany; fkeskin@ukaachen.de

**Keywords:** schizophrenia, vitamin D, anticholinergic drugs, antipsychotics, pharmacokinetics, cognition, processing speed, BACS

## Abstract

Vitamin D deficiency is a frequent finding in schizophrenia and may contribute to neurocognitive dysfunction, a core element of the disease. However, there is limited knowledge about the neuropsychological profile of vitamin D deficiency-related cognitive deficits and their underlying molecular mechanisms. As an inductor of cytochrome P450 3A4, a lack of vitamin D might aggravate cognitive deficits by increased exposure to anticholinergic antipsychotics. This cross-sectional study aims to assess the relationship between 25-OH-vitamin D-serum concentrations, anticholinergic drug exposure and neurocognitive functioning (Brief Assessment of Cognition in Schizophrenia, BACS, and Trail Making Test, TMT) in 141 patients with schizophrenia. The anticholinergic drug exposure was estimated by adjusting the concentration of each drug for its individual muscarinic receptor affinity. Using regression analysis, we observed a positive relationship between vitamin D levels and processing speed (TMT-A and BACS Symbol Coding) as well as executive functioning (TMT-B and BACS Tower of London). Moreover, a negative impact of vitamin D on anticholinergic drug exposure emerged, but the latter did not significantly affect cognition. When other cognitive items were included as regressors, the impact of vitamin D remained only significant for the TMT-A. Among the different cognitive impairments in schizophrenia, vitamin D deficiency may most directly affect processing speed, which in turn may aggravate deficits in executive functioning. This finding is not explained by a cytochrome P450-mediated increased exposure to anticholinergic antipsychotics.

## 1. Introduction

Schizophrenia is a severe mental disorder characterized by multiple disturbances of perception, emotion and cognition [1]. It represents a multifactorial disease with high heritability, typically manifests in early adulthood and is associated with functional impairment, reduced life expectancy and socioeconomic burden [2]. Symptoms can be categorized into positive symptoms such as delusions and hallucinations, negative symptoms such as affective flattening and social withdrawal as well as cognitive symptoms [1].

Cognitive dysfunction is a core element of schizophrenia and one of the major factors contributing to long-term disability in this patient cohort [3]. Cognitive deficits in schizophrenia particularly comprise deficits in processing speed, attention/vigilance, social cognition, verbal learning, visual learning, as well as working memory, reasoning/problem solving and other aspects of executive function (such as abstract thinking and cognitive flexibility) [4,5,6]. However, current treatment options for this symptom domain are still insufficient. Remarkably, cognitive deficits typically manifest early in the disease course—i.e., before the diagnostic criteria are fulfilled—and persist despite successful pharmacological treatment of positive symptoms [7]. Neurobiological correlates of cognitive dysfunction may be impairments of dorsolateral prefrontal cortex function and its interactions with other brain regions [5], altered hippocampal activity [8] and bottom-up consequences of early sensory processing deficits [9,10,11,12,13]. Given the multifactorial nature of schizophrenia and its heterogeneous clinical phenotype, several factors may be ethiopathogenetically relevant for the emergence of cognitive dysfunction in this disorder with their individual contributions varying from patient to patient [14,15]. These factors may also include iatrogenic effects such as the prescription of antipsychotics with high anticholinergic potency [16].

Schizophrenia and other severe mental disorders are associated with lower levels of vitamin D [17]. Given the abundance of vitamin D-receptors in the human body, a lack of this hormone may have consequences going far beyond its well-known role in calcium homeostasis. Indeed, vitamin D deficiency is linked to a generally higher mortality with a significant contribution of cardiovascular diseases and cancer [18]. Therefore, it may be one of the factors leading to reduced life expectancy in this vulnerable patient cohort [19]. Moreover, there is growing evidence for vitamin D deficiency as a risk factor for the development of schizophrenia [7] and its aggravating impact on psychopathology [20,21]. In particular, neonatal vitamin D deprivation is associated with increased risk of developing schizophrenia [22,23]. Accordingly, as adults, rodent models of developmental vitamin D deficiency exhibit a phenotype mimicking some aspects of schizophrenia and show altered dopaminergic neurotransmission [24]. In contrast, in rodent models of adult vitamin D deficiency, there is evidence for a predominant dysfunction of glutamatergic and GABAergic circuits [25,26] and affected animals particularly show cognitive deficits [27,28]. Similarly, a growing number of human studies suggest a contribution of vitamin D deficiency to cognitive deficits in adult patients with schizophrenia and also other mental disorders [29]. However, there is limited knowledge about the clinical profile of vitamin D-related cognitive deficits and their underlying molecular mechanisms. Converging evidence suggests that vitamin D reduces exposure to many different drugs—including antipsychotics [30]—particularly by induction of the Cytochrome P450 (CYP) isoenzyme 3A4 [31,32,33]. Accordingly, vitamin D deficiency might lead to a decreased elimination, i.e., an increased exposure to anticholinergic antipsychotics, which in turn may increase cognitive dysfunction in this patient cohort. In the present study, we therefore aimed to characterize the neuropsychological profile of cognitive deficits in schizophrenia related to vitamin D deficiency and address the potential contribution of a vitamin D-mediated reduction of anticholinergic drug exposure.

## 2. Material and Methods

### 2.1. Participants and Neuropsychological Assessment

Serum concentrations of 25-OH-vitamin D and neuropsychological assessments were obtained from 141 in- and out-patients with schizophrenia. Sociodemographic and clinical characteristics of the patients are given in Table 1. For the different neuropsychological tests, the number of patients who completed them varied slightly (between 130 and 139; see Table 2). In- and out-patients with schizophrenia were recruited at the Department of Psychiatry, Psychotherapy and Psychosomatics of RWTH Aachen University Hospital and four academically associated psychiatric hospitals (Alexianer Hospital, Aachen; ViaNobis Gangelt; LVR Klinik Langenfeld, LVR Klinikum Düsseldorf). The data were obtained in the framework of an interventional brain imaging study with preregistration (NCT02435095). The study was approved by the ethics committee of the North Rhine medical association (AEKNO) and the ethics committee of the RWTH Aachen University Hospital (EK 156/16). We obtained written informed consent from all participants after a complete description of the study. The intended sample size with respect to the primary outcome (gray matter change) of 574 patients could not be achieved and therefore the trial was stopped. All data presented here were obtained before any intervention.

The study employed the following inclusion criteria: diagnosis of schizophrenia according to DSM-5, age of 18–65 years, written declaration of consent, subjects being contractually and mentally capable to attend the medical staffs’ orders and MRI capability. Exclusion criteria comprised: relevant somatic diseases, which could have an impact on the conduct of the study based on clinical judgement of the treating physician (e.g., epilepsy, cancer), prior to insufficiently documented drug therapy with antipsychotics, magnetic metals in and on the body, cardiac pacemakers and body piercings, pregnancy or lactation, hospitalization of the patient ordered by the court or public authorities, relationship of dependence or employment to sponsor or investigator and simultaneous participation in another clinical trial. Trained psychiatrists confirmed the diagnosis of schizophrenia according to DSM 5 criteria using the structured clinical interview for DSM disorders (SCID) and performed the clinical ratings and neuropsychological assessments. As a structured interview, the SCID was used to minimize interviewer bias. For neuropsychological assessments, the Brief Assessment of Cognition in Schizophrenia (BACS) as well as the Trail Making Test A (TMT-A) and B (TMT-B) were employed [34,35,36]. The BACS is a cognitive battery that was designed to assess multiple cognitive domains affected in schizophrenia in relatively a short time (about 30 to 40 min). It contains seven tests in total, assessing verbal memory, working memory (Digit sequencing), semantic (naming of animals) and lexical verbal fluency, processing speed (Symbol coding Test) and motor speed (Token motor test) as well as reasoning and problem solving (Tower of London Test). In both parts of the TMT, study participants have to connect 25 circles distributed over a sheet of paper as quickly as possible without lifting the pen or pencil from the paper. In Part A, the circles are numbered from 1 to 25, and the numbers should be connected in ascending order. In Part B, the circles include both numbers (1–13) and letters (A–L); which again should be connected in an ascending pattern (concerning the size of the number and the alphabetical order of the letters), but alternating between numbers and letters (i.e., 1-A-2-B, etc.).

### 2.2. Quantification of Vitamin D Levels and Anticholinergic Drug Exposure

Within this study, blood samples were taken throughout the year between August 2015 and March 2020. A part of the vitamin D and drug concentrations used for this analysis were already used previously [30]. According to the study protocol, all patients underwent blood sampling for the analysis of vitamin D levels. If patients were already under antipsychotic treatment, we also obtained blood samples for therapeutic drug monitoring. For clinical and organizational reasons, time-points of blood sampling slightly varied from patient to patient. Ideally, for therapeutic drug monitoring, blood samples should be collected just before intake (providing trough levels) and at steady-state conditions of the respective drug (i.e., after more than 4 elimination half-lives under the same dose). In case these conditions were not met, hospital charts were reviewed to identify accurate drug concentrations obtained during the clinical routine within a maximum temporal window of 2 months before or after the determination of vitamin D concentrations. Drug concentrations that were still outside the steady state conditions, for which the dose or time of intake could not be determined or which were obtained during the drug absorption phase (i.e., before the expected time of maximum concentration, Tmax) were excluded from the further analysis. If blood samples were not immediately collected before the next drug intake (a typical clinical situation is given for antipsychotics, which are taken as a single dose in the evening, but blood is withdrawn in the morning), expected trough levels (*C_min_*) were calculated using the drug’s half-life (t_1/2_) and the following exponential function [37]:

$$Cmin=C\left(t\right)*e^{-ke\;*\left(tmin-t\right)}$$

with *C*(*t*) as the drug concentration measured at time *t*, *t_min_* as the time at *C_min_*, and *k_e_* as the elimination rate constant (*k_e_* = ln2/t_1/2_). From the final set drug through levels, dose-adjusted drug concentrations were calculated by dividing them by the given daily dose (C/D) [in (ng/mL)/(mg/day)]. If the date of TDM differed from the date of neuropsychological testing, d0, (i.e., drug levels had to be retrieved from the hospital charts), drug levels at d0 were estimated by multiplying the dose-adjusted drug concentrations with the dose given at d0.

From the total sample, 75 quality-controlled serum concentrations of different antipsychotic drugs obtained from 61 patients were available: amisulpride (N = 12), aripiprazole (N = 8), clozapine (N = 9), olanzapine (N = 16), quetiapine (N = 8) and risperidone (N = 22). The remaining patients either were not yet medicated at the time of assessment or serum concentrations did not fulfil our quality criteria. Among those 75 serum levels, 62 were corrected using the equation above.

For aripiprazole, clozapine, olanzapine, quetiapine, and risperidone, serum concentrations of their respective main metabolites were also determined, i.e., dehydroaripiprazole, norclozapine, desmethylolanzapine, norquetiapine, and 9-OH-risperidone. All drug and metabolite concentrations were analyzed in the same laboratory by Liquid Chromatography with tandem mass spectrometry (LC-MS/MS) [38]. For vitamin D levels (25-OH Vitamin D), chemiluminescent immunoassays (CLIA) were applied [39].

To control for potential pharmacokinetic confounding variables, we screened all patients for the co-prescription of drugs with known inducing or inhibiting properties on the major cytochrome P450 isoenzymes CYP1A2, CYP2B6, CYP2C8, CYP2C9, CYP2C19, CYP2D6 (only inhibitors are known) and CYP3A4 according to the suggestions by the US Food and Drug Administration [40]. We identified two patients receiving the CYP1A2-inhibitor fluvoxamine and one patient receiving the CYP2D6-inhibitor fluoxetine as a co-medication, respectively. The latter patient was not included in any pharmacokinetic analysis, as there was no TDM data available meeting our quality criteria. To assess the impact of the two remaining patients who were under co-treatment with fluvoxamine, we conducted a sub-analysis for which we removed these patients (see Section 3).

For the estimation of anticholinergic exposure, drug serum concentrations were first normalized to the upper level of the therapeutic reference range TRRmax. For risperidone, the concentration of the active moiety (parent compound + active metabolite 9-OH-risperidone) was used instead of the pure concentration of the parent compound, as the TRR is typically defined for the active moiety [37]. Normalized concentrations were adjusted for each drug’s anticholinergic potency by division by the drug’s M1 muscarinic receptor dissociation constant Kd and multiplication with the corresponding dissociation constant Kd of the reference substance chlorpromazine, i.e.,

$$C\left(\mathrm{Anticholinergic}\right)=\frac{C\left(\mathrm{Drug}\;x\right)*\mathrm{Kd}\left(\mathrm{Chlorpromazine}\right)}{\mathrm{TRRmax}\left(\mathrm{Drug}\;x\right)*\mathrm{Kd}\left(\mathrm{Drug}\;x\right)}$$

Dissociation constants (Kd) were obtained from the NIMH Psychoactive Drug Screening Program (PDSP) Database [41] Chlorpromazine, which is typically considered the first antipsychotic drug [42] and is often used to compare antipsychotic potency (chlorpromazine equivalent dose) [43], was chosen as a reference substance due to its broad receptor profile including affinity for muscarinic M1 receptors [44]. If a patient received more than one antipsychotic drug, the respective measures of anticholinergic drug exposure were added to each other.

### 2.3. Statistical Analysis

In order to assess the different relationships between the variables of interest, we conducted four main regression analyses:

(1) In the first regression analysis, we assessed the impact of vitamin D on cognition. Thereto, for each of the 10 cognitive items, we separately calculated a bivariate regression model with the respective item as the dependent variable and the 25-OH-vitamin D concentration as the independent variable, yielding 10 regression models in total.
(2) In the second analysis, we investigated the influence of anticholinergic drug exposure on cognition. As in the first analysis, we calculated separate bivariate regression models for each cognitive item, which served as the dependent variable, and the adjusted anticholinergic drug concentration served as the independent variable.
(3) In the third analysis, we assessed the relationship between vitamin D levels (independent variable) and anticholinergic drug exposure (dependent variable) in a single bivariate regression model. To control for potential confounders, we conducted two additional sub-analyses. In the first one, we added the number of cigarettes per day as a second independent variable to the regression model. In the second one, we removed the two patients receiving a co-treatment with the CYP1A2-inhibitor fluvoxamine.
(4) In a stepwise forward regression analysis, we used one of the three following cognitive items as the dependent variables in separate sub-analyses: TMT-A, TMT-B and the Tower of London. The remaining two cognitive items, the number of cigarettes per day as well as the log-transformed vitamin D and anticholinergic levels served as the independent variables. The independent variables with the most significant impact on the respective dependent variable were sequentially added to the model until no further significant improvement of model fit could be achieved.
(5) As a further exploratory analysis, we assessed the impact of vitamin D on an alternative measure of cognition derived from a five-factor model of the positive and negative syndrome scale (PANSS) [45,46]. This five-factor model includes a “positive”, “negative”, “cognitive”, “emotional/depressed” and “excited” factor, with the cognitive factor being composed of item 2 of the positive symptom subscale, item 5 o the negative symptom subscale and item 11 of the general psychopathology subscale (P2N5G11).

For all regression analyses, in order to verify the linear relationship between the dependent variable and the predictors, individual scatter plots were subjected to a curve fitting analysis, which revealed that some variables had to be log-transformed to establish a linear relationship. In such cases, the respective variables were log-transformed before entering the regression analysis (see Section 3). Gaussian distribution and homoskedasticity of residuals were confirmed by inspection of histograms and Q-Q-plots. If more than one predictor was included in the analysis, absence of multicollinearity was verified by variance inflation factor (VIF), which was required to be below 4. For each of the five main analyses, we used one-tailed *p*-values corrected for multiple comparisons using the Bonferroni correction. Patients with missing values were not included in the respective regression model, i.e., there was no imputation of missing values. Statistical analysis and data visualization were performed using SPSS 28 (IBM, Armonk, NY, USA), RStudio (RStudio Team (2021). RStudio: Integrated Development Environment for R. RStudio, PBC, Boston, MA, USA, Available online: http://www.rstudio.com) and GraphPad Prism 5 (GraphPad Software, San Diego, CA, USA).

## 3. Results

**Figure 1:**
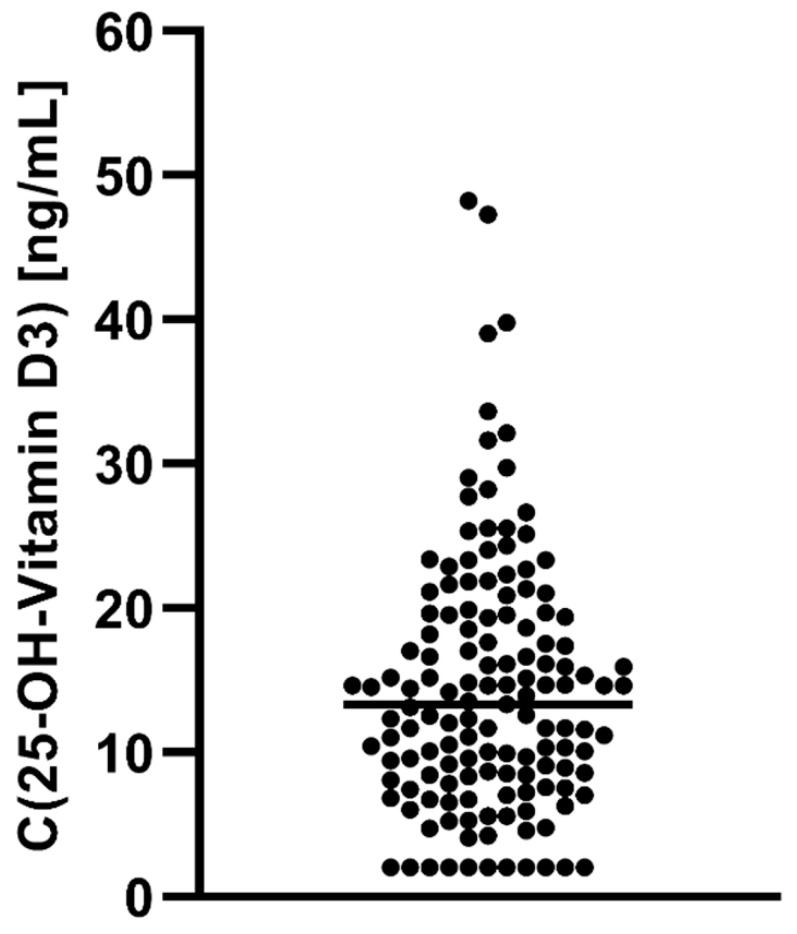
Distribution of 25-OH vitamin D-levels in our sample. Note the high proportions of patients following below the threshold of vitamin D insufficiency (≤20 ng/mL) and deficiency (≤10 ng/mL).

Sociodemographic characteristics of the sample are given in Table 1. Only 22% of patients exhibited sufficient vitamin D levels (>20 ng/mL), i.e., 78% of patients exhibited vitamin D insufficiency (11–20 ng/mL) or deficiency (≤10 ng/mL). For a visualization of the distribution of vitamin D levels, see Figure 1.

**Figure 2:**
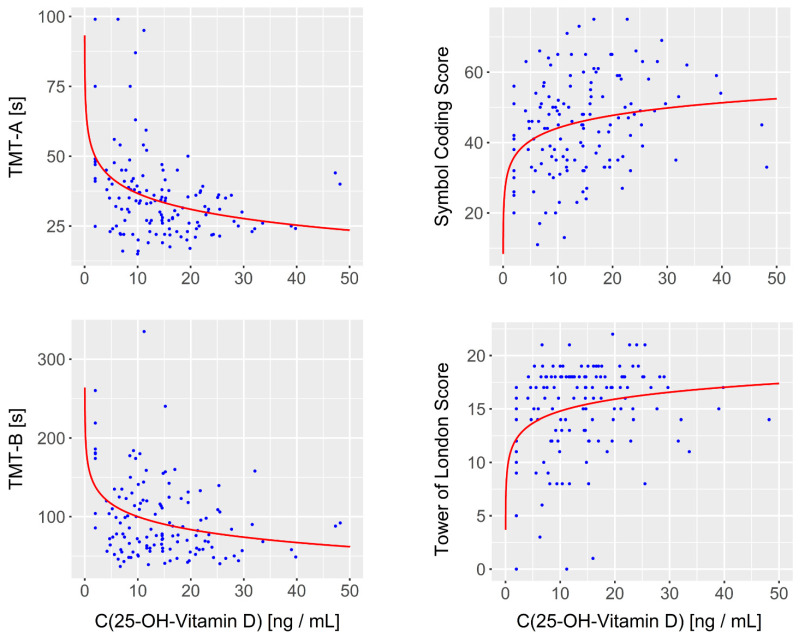
The relationship between 25-OH vitamin D-levels and cognition. Scatter plots and estimated regression curves are shown for the four cognitive items for which vitamin D’s impact remained statistically significant after Bonferroni correction of multiple comparisons. Curve fitting indicated that each of the different cognitive items (*y*-axis) could be best described as a function of the logarithm of the 25-OH-vitamin D-concentration (*x*-axis).

Confirming the primary hypothesis, we observed a general positive relationship between vitamin D levels and cognitive performance; i.e., patients with lower vitamin D levels exhibited more pronounced cognitive impairments (see Table 2). The curve fitting revealed that each of the different cognitive items could be best described as a function of the logarithm of the 25-OH-vitamin D-concentration (see Figure 2). After a Bonferroni correction of multiple comparisons, the impact of vitamin D on four cognitive items remained statistically significant including the TMT-A (*p* < 0.001) and the BACS Symbol Coding Test (*p* = 0.004) as measures of processing speed as well as the TMT-B (*p* < 0.001) and BACS Tower of London Test (*p* = 0.006) as measures of executive functioning with the former operationalizing cognitive flexibility and the latter planning and problem solving. Corresponding Pearson correlation coefficients addressing the correlation between the respective cognitive items and the log-transformed 25-OH-vitamin D-concentration as well as uncorrected *p*-values are provided in Table 2.

Only at a trend level, a negative relationship between the estimated anticholinergic drug levels and each of the cognitive items emerged. Similar to the first regression analysis, a best curve fit could be achieved when applying a logarithmic function. However, even without correction for multiple testing, none of the regression models reached statistical significance. For detailed statistics, see Table 2. Whereas the negative effect of anti-cholinergic substances of cognition is consistent with the data, the significance of such effects seems lesser than the positive vitamin D effects.

**Figure 3:**
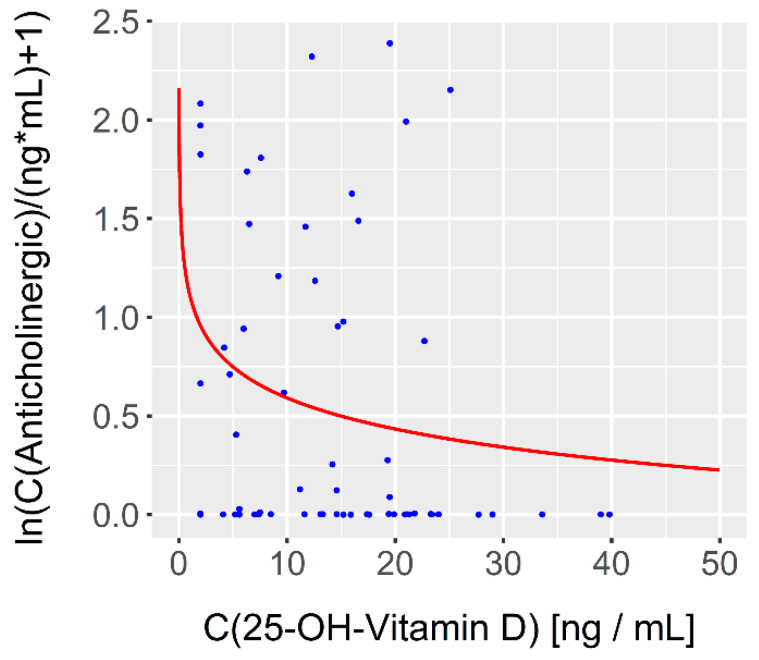
The relationship between 25-OH vitamin D-levels and exposure to anticholinergic antipsychotics. Scatter plot of the association between 25-OH-vitamin-concentration (*x*-axis) and antipsychotic drug concentrations adjusted for anticholinergic potency (*y*-axis). Curve fitting revealed a best fit for a log-transformation of both the dependent and independent variable.

We observed a negative association between 25-OH-vitamin D-concentration and anticholinergic drug exposure. Curve fitting revealed a best fit for logarithmic transformation of both the independent and dependent variable (see Figure 3). This association reached statistical significance (standardized beta = −0.235; *p* = 0.034; N = 61), confirming the pharmacokinetic relationship between vitamin D and (some) anti-cholinergic drugs. Since smoking is known to induce the cytochrome P450 isoenzyme CYP1A2, we subsequently included the number of cigarettes per day as a second predictor to assess the effect of this potential confounder on anticholinergic drug exposure. This covariate did not show any association with the dependent variable (standardized beta = −0.014; *p* = 0.913; N = 61), whereas the effect of vitamin D remained significant (standardized beta = −0.235; *p* = 0.036; N = 61). To control for further pharmacokinetic confounders, for all patients we assessed the prescription of co-medication with known inducing or inhibiting properties on the major cytochrome P450 isoenzymes. We identified two patients who received the CYP1A2-inhibitor fluvoxamine and one patient receiving the CYP2D6-inhibitor fluoxetine as a co-medication, respectively. Since for the latter patient, there was no TDM data meeting our quality criteria available, this patient was not included in the analysis. When excluding the two patients who were under co-treatment with fluvoxamine, the effect of vitamin D on anticholinergic drug levels remained significant (Pearson’s r = −0.256; *p* = 0.025; N = 59). We therefore decided not to exclude these two patients from the further analyses.

For the stepwise regression analysis, we selected the TMT-A, TMT-B, and the Tower of London Test performance as the dependent variables, as those were the only three items—besides the Symbol Coding Test—which remained significant after correction for multiple testing. Since the Symbol Coding Test is a further measure of processing speed—just as the TMT-A—we decided to exclude it from the analysis in order to minimize the number of statistical tests. The sub-analyses revealed that for none of the three investigated cognitive scales, anticholinergic drug exposure or the daily number of cigarettes was included in the model. For the TMT-A, the best model fit could be attained (R^2^ = 0.418) when including the Tower of London Test performance (standardized beta = −0.477; t = −4.434; *p* < 0.001) and the log-transformed 25-OH-vitamin D concentration (standardized beta = −0.319; t = −2960; *p* = 0.003). For the TMT-B, the final model (R^2^ = 0.339) included the TMT-A (standardized beta = 0.352; t = −2.638; *p* = 0.006) and the Tower of London Test (standardized beta = −0.304; t = −2.278; *p* = 0.014). Finally, for the Tower of London Test performance (R^2^ = 0.339), the TMT-A (standardized beta = −0.422; t = −3.389; *p* < 0.001) and the TMT-B (standardized beta = −0.283; t = −2.278; *p* = 0.014) were included in the model.

Thus, after regressing out the respective other tests, only for the TMT-A, the inclusion of log-transformed 25-OH-vitamin D concentration resulted in a significant improvement of model fit. This effect remained also significant after Bonferroni correction (*p*-corrected = 0.045).

**Figure 4:**
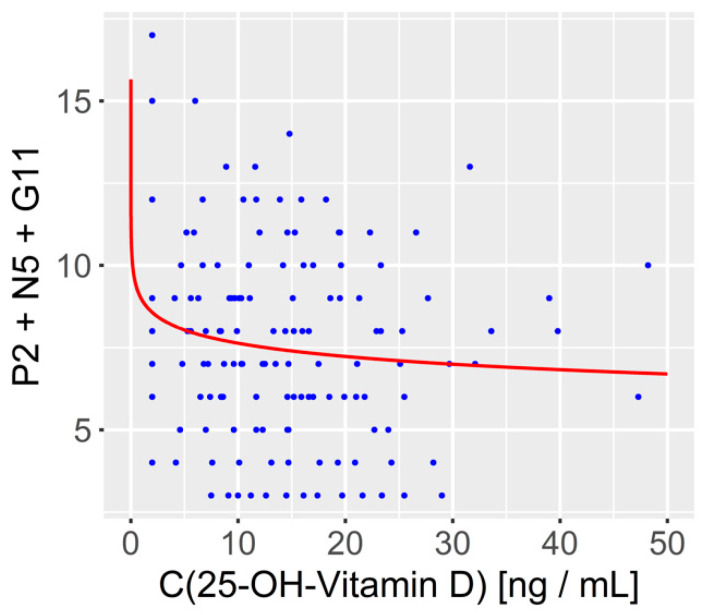
The relationship between 25-OH vitamin D levels and the cognitive component of the PANSS five factor model derived from items P2, N5 and G11 of the PANSS. As for the other cognitive variables, curve fitting indicated that the cognitive component (*y*-axis) could be best described as a function of the logarithm of the 25-OH-vitamin D concentration (*x*-axis).

Further, we investigated the relationship between vitamin D concentration and an alternative measure of cognition, namely the cognitive component of a five-factor model of the positive and negative syndrome scale (PANSS) [45,46]. Again, lower 25-OH-vitamin D levels were associated with greater cognitive impairment, and curve fitting revealed a logarithmic relationship between the dependent (i.e., the cognitive) variable and the independent variable (vitamin D concentration). (Standardized beta = −0.144; *p* = 0.046; N = 139) (see Figure 4).

## 4. Discussion

A significant proportion of patients with schizophrenia suffer from vitamin D deficiency, which may contribute to somatic comorbidity and psychopathology, particularly cognitive symptoms. The present study thus confirmed prior evidence for vitamin D deficiency as a factor contributing to neurocognitive dysfunction in schizophrenia and provided a characterization of the neuropsychological profile of vitamin D-deficiency-related cognitive deficits. We detected a strong association between vitamin D serum concentrations and processing speed as well as executive functions in patients suffering from schizophrenia. However, a stepwise regression analysis revealed that vitamin D deficiency most directly affected processing speed, while its impact on executive functioning may be better explained as a consequence of the former effect, i.e., its effect on processing speed. Cognitive dysfunction in schizophrenia comprises a well-defined set of cognitive domains, including processing speed, attention/vigilance, visual and verbal learning, and social cognition as well as working memory, reasoning/planning and other executive functions [4]. There is still controversy about the existence of a hierarchy of the different cognitive symptom domains and their causal relationship. However, several studies suggest a pivotal role of deficits in processing speed [47], which may contribute to other cognitive deficits such as working memory deficits and executive dysfunction [48]. Interestingly, our present findings suggest that vitamin D deficiency primarily affects this important cognitive domain. Previous studies addressing the neuropsychological profile of cognitive deficits related to vitamin D deficiency have yielded inconclusive results: In a cross-sectional study assessing cognitive performance in 20 patients with first episode schizophrenia and 20 healthy controls, vitamin D deficiency was associated with lower scores of a summary measure of different cognitive tests in patients with schizophrenia, only [49]. For the individual tests, only verbal fluency was significantly correlated with vitamin D levels, but not processing speed. However, insufficient power due to the small sample size (N = 20) may have biased the results. Based on the relationship between vitamin D and the TMT-A (yielding the highest effect size), a post hoc power analysis of our own data indeed suggests a minimum required sample size of 40 patients given an expected power of 80% and a one-tailed alpha-level of 0.05. For the different cognitive tests that were significantly correlated with vitamin D concentrations in our own dataset, the post hoc power analysis estimated a power of 99.8% for the TMT-A, 99.2% for the TMT-B, 96.2% for the BACS Symbol Coding Test and 95.3% for the BACS Tower of London Test, respectively, given the respective sample sizes and a one-tailed alpha-level of 0.05. Accordingly, in a larger sample of 225 patients with psychotic disorders, Nerhus et al. observed that a low vitamin D status was significantly associated with decreased processing speed and verbal memory [50]. Similar to the present study, the strongest association was observed for processing speed. In a randomized, double-blind, placebo-controlled clinical trial, 47 patients with therapy-resistant schizophrenia and low vitamin D levels were randomly assigned to a vitamin D supplementation or placebo group [51]. After eight weeks, the vitamin D group demonstrated a significant increase in vitamin D levels and a trend towards improved cognition, particularly for attention and verbal memory. Notably, the authors applied the Montreal Cognitive Assessment (MOCA), which does not include an explicit test for processing speed [52]. Larger clinical trials are desirable to draw further conclusions on the effectiveness of vitamin D supplementation on cognitive symptoms in schizophrenia. As a neural correlate of improved cognitive performance, there is first evidence for an amelioration of hippocampal volume loss in schizophrenia mediated by vitamin D [53], but there is still limited knowledge on the molecular mechanisms of vitamin D’s neurophysiological effects. A potential mechanism that we wanted to address in this study is grounded in vitamin D’s impact on drug metabolism. Indeed, vitamin D has been demonstrated to increase metabolism and elimination of many different drugs including antipsychotics [30]—particularly CYP3A4 substrates. Since there is converging evidence for a negative impact of antipsychotics with high anticholinergic potency on cognition [16], we assessed whether the effect of a low vitamin D status on cognition in schizophrenia might be mediated by a reduced metabolism of anticholinergic antipsychotics. Several in vitro studies demonstrated CYP3A4 induction by vitamin D in different cell lines including primary human hepatocytes [54,55,56]. Human in vivo studies revealed that the supplementation of vitamin D is associated with increased elimination of the statin and CYP3A4 substrate atorvastatin [33]. Moreover, blood concentrations of the immunosuppressants tacrolimus and sirolimus—both of which are substrates of CYP3A4—show a cyclic seasonal variation, which is anti-correlated to the well-known seasonal variation of vitamin D levels [32]. Similarly, intestinal CYP3A4 expression was demonstrated to be predicted by genetic polymorphisms of the vitamin D receptor [57]. beyond CYP3A4, there is preliminary evidence suggesting that vitamin D also has inducing properties on the isoenzymes CYP2B6 and CYP2C9 —with probably minor quantitative contribution, though [54] as well as *p*-glycoprotein (*p*-gp), a renal efflux pump of xenobiotics [58]. Accordingly, vitamin D deficiency might lead to a decreased elimination, i.e., an increased exposure to anticholinergic antipsychotics, which in turn may increase cognitive dysfunction in this patient cohort. However, even though we observed a significant negative relationship between vitamin D levels and the exposure to anticholinergic antipsychotics, this finding could not explain the robust effects of vitamin D on the cognition observed in this study. Several animal studies have suggested a neurotrophic effect of vitamin D promoting neurogenesis and enhancing synaptic function in the hippocampus [59,60]. Accordingly, a human study suggested an amelioration of hippocampal volume loss in schizophrenia mediated by vitamin D (see above) [53]. Cognitive dysfunction in schizophrenia may also be related to inflammatory processes [61,62,63]. Indeed, increased serum concentrations of C-reactive protein (CRP), a peripheral marker of inflammation, were associated with worse cognitive performance in patients with schizophrenia [64]. Notably, vitamin D has been found to regulate the production of proinflammatory cytokines and the proliferation of proinflammatory cells, respectively [65]. Accordingly, such anti-inflammatory properties may represent a mechanism that might explain its potential benefits for cognition in schizophrenia. Statins may constitute a further candidate drug group to modulate inflammatory processes in schizophrenia [66]. Other potential molecular targets of pro-cognitive pharmacotherapy may be N-Methyl-D-Aspartate (NMDA) receptors, metabotropic glutamate receptors and the kynurenine pathway [67,68,69]. Cognitive deficits in schizophrenia are likely multifactorial and may require different treatment approaches for the individual patients. The identification of pathophysiologically specific molecular markers (e.g., [70,71]) obtained from easily accessible biomaterial or brain imaging endophenotypes and combined with machine learning algorithms may serve as a basis for the establishment of precision medicine in psychiatry [72].

Several test batteries have been employed to study cognitive deficits in schizophrenia (for an overview see [73]). An ideal test battery should cover most cognitive domains affected in schizophrenia within an appropriate time frame, which should be tolerable for most patients and economic for staff members administering the tests. Among the different test batteries that were used in the literature, the MATRICS Consensus Cognitive Battery (MCCB) and the Brief Assessment of Cognition in Schizophrenia (BACS) represent two well-validated and reliable instruments meeting the abovementioned criteria. The BACS, which was used in the present study, is particularly short (around 30 min for completion) while covering most of the cognitive domains that are impaired in patients with schizophrenia. It comprises seven tests in total, examining verbal memory, working memory (Digit sequencing), semantic (naming of animals) and lexical verbal fluency, processing speed (Symbol coding Test) and motor speed (Token motor test) as well as reasoning and problem solving as an aspect of executive function (Tower of London Test). It was shown to be as sensitive to cognitive impairment in schizophrenia as more extensive test batteries. Since the battery is specifically designed to measure treatment-related changes of cognitive symptoms, it provides alternate forms for some of the tests in order to minimize practice effects. Moreover, it is available in nine languages and norms are also available. In the present study, besides the BACS, we additionally administered The Trail Making Test (TMT)-A and -B, as both tests require a minimum time for completion but provide an additional measure of processing speed (TMT-A) as well as a measure of cognitive flexibility (TMT-B). The TMT, which was originally introduced as a part of the Army Individual Test Battery [74], represents one of the most popular neuropsychological tests employed by many different test batteries [75] and patients with schizophrenia have been demonstrated to exhibit significant performance deficits for both tests [76].

As stated above, a good alternative to the set of tests used in this study (BACS and TMT) may be the MCCB. It comprises 10 tests selected by experts within the framework of the NIMH Measurement and Treatment Research to Improve Cognition in Schizophrenia (MATRICS) based on more than 90 tests nominated for inclusion [77]. Notably, the TMT-A and the BACS Symbol Coding Test (both assessing processing speed) as well as an animal naming test comparable to the one which is part of the BACS are included in this battery. The remaining seven tests examine attention/vigilance (Continuous Performance Test—Identical Pairs), working memory (WMS–III Spatial Span; University of Maryland Letter-Number Span), verbal memory (Hopkins Verbal Learning Test—Revised), visual memory (Brief Visuospatial Memory Test—Revised), reasoning and problem-solving (Neuropsychological Assessment Battery—Mazes) as well as social cognition (Mayer–Salovey–Caruso Emotional Intelligence Test—Managing Emotions). Accordingly, while there is a substantial overlap between the assessed cognitive domains and administered tests of the MCCB and the set of tests employed in this study, the MCCB provides tests for attention/vigilance, visual memory, and social cognition that are not assessed explicitly by the BACS or TMT, whereas our set of tests provides additional measures of motor skills (Token motor test) and cognitive flexibility (TMT-B). Another advantage of our approach is the lower amount of time required for completion (around 30 to 40 min as compared to 60 min for the MCCB). Future studies should also investigate the relationship between vitamin D and tests of attention/vigilance, visual memory and social cognition, as provided by the MCCB.

## 5. Limitations

A major limitation of the present study is its cross-sectional and non-interventional nature. Accordingly, the correlations reported in the present study may in principle reflect pure epiphenomena, but not necessarily a causal relationship. Moreover, the true causal relationship may also be reverse, i.e., cognitive deficits may also lead to lower vitamin D levels. Due to the inability to perform everyday activities, patients with cognitive deficits may spend less time outdoors and therefore may be less exposed to sunlight. This hypothesis has been also stated for elderly persons with cognitive deficits. However, according to our hypothesis, animal studies [59,60,78,79] and first randomized controlled clinical trials [51] have provided some preliminary evidence for a direct causal impact of vitamin D on cognition, Further randomized controlled clinical trials (RCTs) comparing the effects of vitamin D supplementation in comparison to a placebo group are warranted. Ideally, such studies should apply therapeutic drug monitoring during the course of the treatment in order to control for vitamin D’s negative impact on antipsychotic drug exposure. From a more preventive perspective, screening for vitamin D deficiency and supplementation studies may be also relevant for persons who are at a high risk for schizophrenia [80].

Our inclusion and exclusion criteria may have caused some degree of selection bias. Such as many other studies on patients suffering from severe mental disorders, for ethical and legal reasons, we only included subjects being contractually and mentally capable to attend the medical staffs’ orders and understand the study procedure. Moreover, we excluded patients whose hospitalization was ordered by the court or public authorities. As a consequence, patients with less severe psychopathology may be overrepresented in our study cohort. Moreover, due to the fact that the study was part of a larger brain imaging trial, we only considered patients who met the MRI safety criteria which are—however—not relevant for the data that were the basis of this study.

## 6. Conclusions

Cognitive dysfunction is a core symptom domain of schizophrenia associated with long-term disability, but limited treatment options. In the present study, we observed a significant association between serum concentrations of vitamin D—which are insufficient in many patients with schizophrenia—and cognitive performance, particularly processing speed. This relationship could not be explained by the negative impact of vitamin D on the exposure to anticholinergic antipsychotics—given its inducing effects on cytochrome P450 isoenzymes—particularly CYP3A4. Considering vitamin D’s well-established effects on physical health, the growing evidence for its effects on mental health and cognition as well as the frequency of vitamin D insufficiency in schizophrenia, screening for vitamin D insufficiency and its compensation by supplementation may be beneficial for this vulnerable patient cohort.

## Figures and Tables

**Table 1:** Sociodemographic and clinical characteristics of the sample.

Characteristic	Mean	Std
**Biometrics**		
Age [Years]	33.1	11.4
C (25-OH-Vitamin D3) [ng/mL]	14.5	8.9
Duration of disease	4.9	7.1
**Cognitive Performance**		
TMT-A [s]	35.3	15.3
TMT-B [s]	95.9	49.6
BACS—Verbal Memory	37.8	12.5
BACS—Working Memory: Correct Answers	17.6	4.4
BACS—Working Memory: Longest Sequence	6.3	1.6
BACS—Motor Speed	64.0	17.6
BACS—Fluent Speech Category	18.1	5.2
BACS—Fluent Speech Letter	20.1	7.9
BACS—Symbol Coding	44.9	13.5
BACS—Tower of London	15.0	4.2
**Positive and Negative Syndrome Scale (PANSS)**		
Positive Symptoms	16.1	6.9
Negative Symptoms	17.8	6.6
Global Symptoms	32.8	10.9
	**N**	**%**
**Gender**		
Female	40	28.4
Male	101	71.6

BACS = Brief Assessment of Cognition in Schizophrenia.

**Table 2:** Correlation analysis.

		C(25-OH-Vitamin D) ^a^	C(Anticholinergic) ^a^
TMT−A (in s)	Pearson’s r	** −0.373	0.169
	*p*-value	<0.001	0.097
	N	133	61
TMT−B (in s)	Pearson’s r	** −0.336	0.174
	*p*-value	<0.001	0.092
	N	130	60
Verbal Memory	Pearson’s r	0.175	−0.124
	*p*-value	0.020	0.173
	N	139	60
Working Memory Correct Answers	Pearson’s r	0.131	−0.048
	*p*-value	0.062	0.358
	N	139	60
Working Memory Longest Sequence	Pearson’s r	0.204	−0.022
	*p*-value	0.008	0.434
	N	139	60
Motor Speed	Pearson’s r	0.107	−0.122
	*p*-value	0.108	0.184
	N	136	57
Fluent Speech Category	Pearson’s r	0.195	0.001
	*p*-value	0.011	0.496
	N	137	59
Fluent Speech Letter	Pearson’s r	0.136	−0.044
	*p*-value	0.056	0.370
	N	138	59
Symbol Coding	Pearson’s r	** 0.280	−0.070
	*p*-value	<0.001	0.299
	N	139	59
Tower of London	Pearson’s r	** 0.274	−0.103
	*p*-value	<0.001	0.220
	N	137	59

** Correlation remains statistically significant after Bonferroni correction for multiple testing. Given *p*-values in the table are uncorrected. Corrected *p*-values are given in the main text. ^a^ C (25-OH-vitamin D) and C (anticholinergic) were log-transformed, as the curve fitting revealed a logarithmic relationship.

## Data Availability

Data are stored at RWTH Aachen University hospital. The data are not publicly available due to privacy and ethical restrictions.
